# Membrane Attack Complex in Myocardial Ischemia/Reperfusion Injury: A Systematic Review for Post Mortem Applications

**DOI:** 10.3390/diagnostics10110898

**Published:** 2020-11-02

**Authors:** Cristina Mondello, Elvira Ventura Spagnolo, Luigi Cardia, Daniela Sapienza, Serena Scurria, Patrizia Gualniera, Alessio Asmundo

**Affiliations:** 1 Department of Biomedical and Dental Sciences and Morphofunctional Imaging, University of Messina, via Consolare Valeria, 1, 98125 Messina, Italy; dsapienza@unime.it (D.S.); sscurria@unime.it (S.S.); patrizia.gualniera@unime.it (P.G.); aasmundo@unime.it (A.A.); 2 Section Legal Medicine, Department of Health Promotion Sciences, Maternal and Infant Care, Internal Medicine and Medical Specialties (PROMISE), University of Palermo, Via del Vespro, 129, 90127 Palermo, Italy; 3 IRCCS Centro Neurolesi Bonino-Pulejo, 98100 Messina, Italy; luigicardia1@gmail.com

**Keywords:** myocardial infarction, ischemia/reperfusion injury, complement system, C5b-9, forensic pathology, post mortem investigation

## Abstract

The complement system has a significant role in myocardial ischemia/reperfusion injury, being responsible for cell lysis and amplification of inflammatory response. In this context, several studies highlight that terminal complement complex C5b-9, also known as the membrane attack complex (MAC), is a significant contributor. The MAC functions were studied by many researchers analyzing the characteristics of its activation in myocardial infarction. Here, a systematic literature review was reported to evaluate the principal features, advantages, and limits (regarding the application) of complement components and MAC in post mortem settings to perform the diagnosis of myocardial ischemia/infarction. The review was performed according to specific inclusion and exclusion criteria, and a total of 26 studies were identified. Several methods studied MAC, and each study contributes to defining better how and when it affects the myocardial damage in ischemic/reperfusion injury. The articles were discussed, focusing on the specificity, sensibility, and post mortem stability of MAC as a marker of myocardial ischemia/infarction, supporting the usefulness in routine post mortem investigations.

## 1. Introduction

The complement system contribution to myocardial damage due to a severe reduction of blood flow has been studied by many researchers who described its critical role in myocardial ischemia/reperfusion injuries [1,2,3]. The coronary atherosclerotic disease is responsible for the majority of ischemic events and represents the most common cause of sudden cardiac death (SCD) [4,5,6], followed by cardiomyopathies, myocarditis, valvular disease, and channelopathies [7,8,9,10].

Prolonged myocardial ischemia causes an irreversible loss of function associated with cell death, appearing in histological findings as coagulative necrosis, the pathognomonic sign of myocardial infarction. On the other hand, the restoration of blood flow within a critical period of 20 min from the onset of ischemic insult is associated with the resolution of the initial structural and functional tissue change. After this temporal window, the ischemic damage becomes irreversible, resulting in a paradoxical state in which the tissue damage could be worsened by reperfusion. In fact, the reintroduction of flow mediates a massive inflammatory response increasing the extent of tissue damage beyond due to ischemia [11]. In particular, ischemic/reperfusion insult induces cellular injury and death to the tissue components (cardiomyocytes, fibroblasts, endothelial cells, and interstitium) and, consequently, determines an acute proinflammatory response which is mediated by complement activation, reactive oxygen species (ROS) production, and damage-associated molecular patterns (DAMPs) [12]. DAMPs (i.e., ATP and HMBGB1) promote the formation of inflammasomes by interacting with pattern recognition receptors (PRRs) such as Toll-like receptors (TLRs) [13,14]. These reactions are associated with the release of several proinflammatory mediators (i.e., IL-1, IL-6, MCP-1/CCR-2, and CCL5), inducing the recruitment of inflammatory cells (neutrophils, monocytes, macrophages, and T and B lymphocytes) [15,16,17].

The complex mechanism of myocardial ischemia/reperfusion injury is strongly correlated to the complement system, which has also been described as a contributing factor in the destabilizing process of atherosclerotic plaque determining the coronary occlusion supporting the tissue ischemia [18,19,20]. It follows that the complement system should be involved in the pathophysiological process of SCD due to the coronary atherosclerotic disease being able to contribute to both the dynamic variations of the plaques and the anatomic and functional alteration of myocardial tissue [21]. 

The research aimed to define the role better and the function of the complement system and membrane attack complex (MAC) in myocardial ischemic damage with clinical and therapeutic purposes had a significant impact also on the forensic field having represented the substrate useful to improve the tools promoting an effective post mortem diagnosis in cases of SCD related to early myocardial ischemia (EMI).

EMI diagnosis can be problematic for forensic pathologist because the gross analysis and the routine hematoxylin and eosin (H&E) staining of the heart provide no specific signs [22]; in fact, the coagulative necrosis requires several hours, thus, when death occurs after a short period (from minutes to few hours: ≤ 6–8 h) from the onset of ischemic injury, this pathognomonic sign is lacking [23]. Therefore, during the past years, several studies have been carried out to suggest investigations filling the gaps of post mortem routine analysis, highlighting the usefulness to examine the immune inflammatory and cellular factors that are involved in EMI. That is how the integration of clinical and forensic research has allowed deepening the knowledge on MAC in ischemic myocardial damage, highlighting remarkable evidence.

On the basis of a critical analysis of the literature on the complement system and particularly on the MAC role in myocardial ischemia/infarction, the present review aims to analyze the data emerging from pathophysiological/clinical and forensic studies to summarize all the principal features, advantages and limits regarding the use of these molecules in post mortem setting.

### Activation Pathways and Effects of Complement System in Myocardial Ischemia/Reperfusion Injury

**Figure 1:**
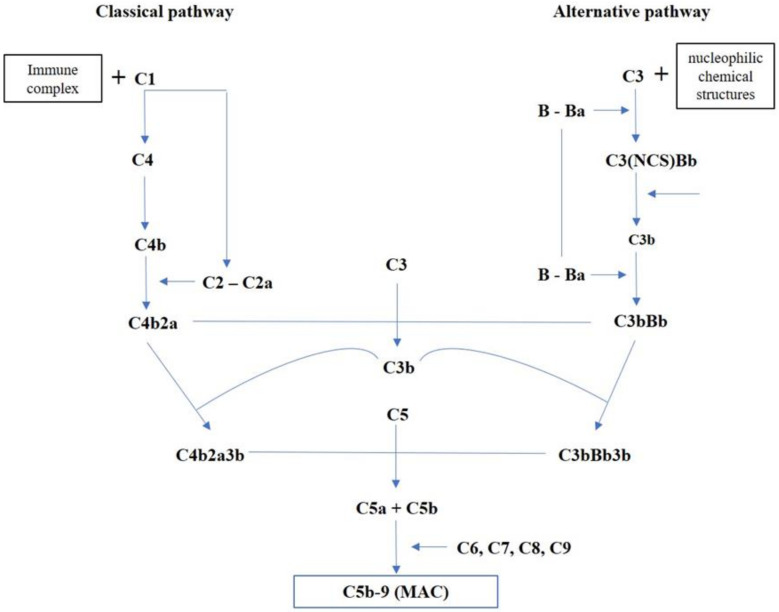
Overview of complement activation pathways.

The evidence on myocardial ischemia/reperfusion damage shows that complement activation, occurring in response to DAMPs and the release of cardiomyocytes contents, is mediated by both the classical and alternative pathways (Figure 1), along with the lectin pathway’s contribution, leading to deposition of MAC C5b-9 [24]. The classical pathway is activated primarily by the immune complex formation and, therefore, is inactive until an antigen interacts with the body; it starts by the interaction between the immune complex and C1. The alternative pathway depends on nucleophilic chemical structures expressed from a target surface that is bound by C3b derived from the C3 cleavage continuously occurring in the fluid phase. Both classical and alternative pathways lead to the formation of C4b2a and C3bBb, respectively, acting as C3 convertases; this represents the point of intersection for the two pathways resulting in the activation of C5b-9 [25].

**Figure 2:**
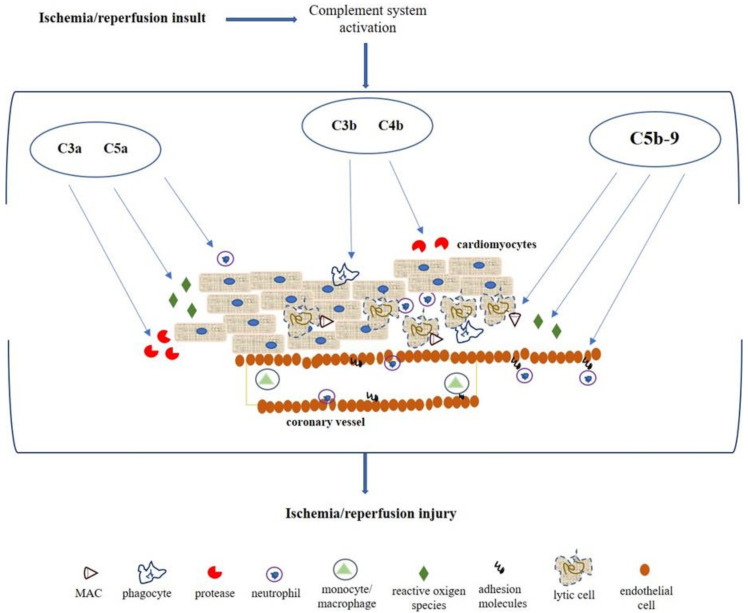
Pathophysiological mechanism mediated by complement system after myocardial ischemia/reperfusion insult. C3a and C5a act as anaphylatoxin promoting the attraction and action of neutrophils, stimulating the reactive oxygen species production and protease release. C3b and C4b mediate opsonization and phagocytosis. MAC is involved in cellular lysis, endothelial expression of adhesion molecules (i.e., E-selectin, ICAM-1, and VCAM-1) for migration and activation of neutrophils.

Many of the proteins activated by the complement system pathways mediate different actions, which lead to the inflammatory response enhancement by the downstream release of proinflammatory cytokines and leukocytes, amplifying tissue damage (Figure 2). The anaphylatoxins C3a and C5a appear the most potent chemotactic factors associated with neutrophils attraction and aggregation, chemotaxis, and cytotoxic activity, also providing a trigger for the release of reactive oxygen metabolites and protease [26,27]. C3b and C4b are involved in opsonization and phagocytosis, promoting the close interaction between the phagocyte and the cell or coated particle, facilitating ingestion, or if the opsonized particle is too large, degranulation that results in the extracellular release of proteases and toxic oxygen metabolites [12,28].

The deposition of MAC is considered one of the most important features of complement activation in myocardial ischemia/reperfusion injury, promoting cellular lysis and amplification of the inflammatory response [29]. Several studies report the MAC involvement in the endothelial activation, resulting in cell adhesion molecules and the release of chemotactic and activating factors contributing to migration and activation of neutrophils [30,31]. Particularly, MAC facilitates the adherence of neutrophils by increasing the expression P-selectin in endothelial cells as well as the secretion of Von Willebrand factor; it also promotes the expression of other cell adhesion molecules such as intercellular adhesion molecule 1 (ICAM-1), vascular cell adhesion molecule 1 (VCAM-1), and Endothelial-selectin (E-selectin) [30,32]. Moreover, these activated cells release several factors such as IL-8, monocyte chemotactic protein 1 (MCP-1), or leukotriene B4 (LTB4) and platelet-activating factor (PAF) that are involved both in the neutrophil recruitment and activation, and contributing to the vascular permeability and cellular edema increase [29,33].

## 2. Materials and Methods 

The authors analyzed the data on complement components and C5b-9 complex in myocardial ischemia/reperfusion injury useful for post mortem diagnosis of early myocardial ischemia. In particular, findings on methods for marker analysis, expression characteristics, the timing of expression, and post mortem stability, were collected.

### 2.1. Search Strategy 

Bibliographic research has been conducted employing the PubMed and Scopus database. The search included articles between January 1990 and September 2020, using the key term firstly “C5b-9 complement complex” to expand the results related to the topic, and then in combination with: “myocardial infarction”, “myocardial ischemia”, “forensic”, “myocardial immunohistochemistry”, “genetics”, and “biochemistry”.

The rule of thumb of articles selection, performed independently by two researchers, was the identification in the titles and/or the abstracts of words/concepts indicating the analysis and the application of complement components and C5b-9 complex in myocardial tissue damage due to ischemia, useful for post mortem investigation. Thus, research articles with clinical/therapeutic and forensic/pathological purposes were read entirely if the abstract indicated that the article potentially met the inclusion criteria.

The articles main inclusion criteria were: English language, year of publication (from 1990 to September 2020), evidence on complement system and MAC activity resulting in the early phase of ischemic injury and/or reperfusion damage. Review articles and editorial comments were excluded.

### 2.2. Study Selection

**Figure 3:**
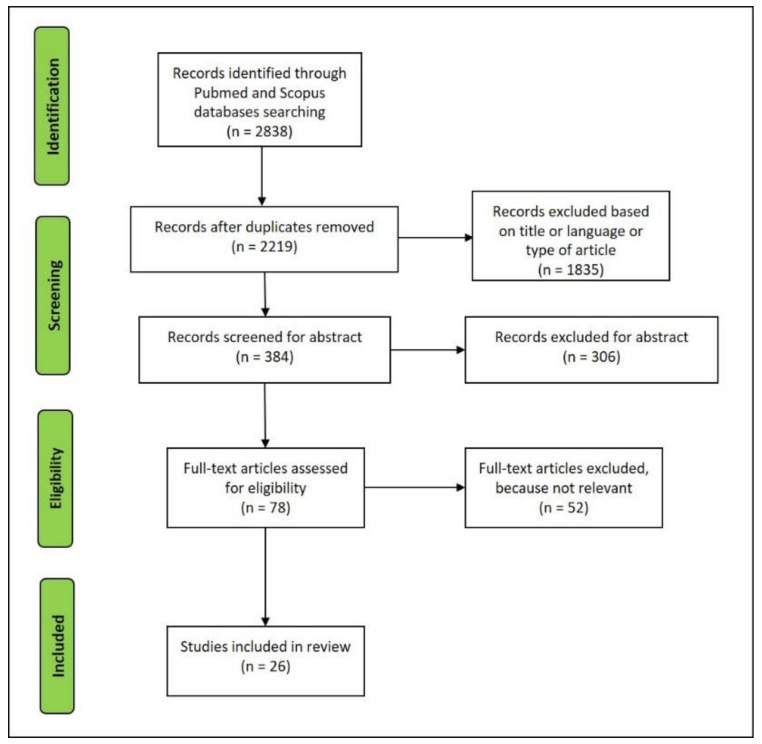
Flowchart of the studies retrieved for the review.

The article selection was performed following the process reported in Figure 3. After duplicates were removed, the paper written in the English language was first screened by title and abstract to obtain the eligible full-texts for reading. Then, the articles entirely read that did not deal with the topic were excluded. 

The articles considered relevant were analyzed in-depth by two investigators independently, focusing on the methods used, the characteristics of complement factors and C5b-9 expression, the data on timing for factors detection, and post mortem stability. No disagreement on data extraction emerged between investigators.

## 3. Results 

A total of 26 articles were selected for reviewing (Figure 3). These articles summarize the principal features of the complement system in myocardial ischemia/infarction and elucidate the advantages and limits related to the use in post mortem settings. 

### 3.1. Summary of Pathophysiological and Clinical Studies

In 1990, Yasuda et al. [34] reported the increasing of iC3b, C3d, C4d, Ba, Bb, and soluble C5b-9 (s) in plasma of patients with angina pectoris and mostly with acute myocardial infarction, in comparison to the plasma of volunteers. This evidence highlighted the activation of both classical and alternative pathways of the complement system and thus the involvement of the cytolytic form of C5b-9 in ischemic myocardial damage, moreover, assuming that the degree of the activated complement system may affect the myocardial necrotic size.

Hugo et al. [35] performed a study on autopsy specimens of infarcted myocardium to quantify the C5b-9 levels and to analyze the differences between the lytic membrane-bound C5b-9 (m) and the soluble C5b-9 (s) complex (cytolytically inactive). An ELISA analysis was carried out showing a markedly elevated amount of (m)C5b-9 complex together with a raised level of (s)C5b-9 in infarcted myocardium when compared to normal tissue in which the (m)C5b-9 was low, and the (s)C5b-9 appeared in varying levels. Moreover, the increasing of (m)C5b-9 in infarcted tissue resulted in the suggestion that tissue-deposited C5b-9 can depend on both types of complex but with a slight dominance of the membrane form.

Väkevä et al. [36] carried out an immunofluorescence study on the pattern of deposition of some complement components and various membrane complement regulators in subjects died of acute myocardial infarction. The analysis revealed that factors of both classical (C1q, C3c, C3d, and C4) and late (C5, C6, C8, and C9) pathways were present in infarcted tissue belonging from patients died after about 8 h from the ischemic insult, with a prevalence of component for the classical pathway suggesting that the complement activation occurred principally by this way. On the other hand, no antibodies deposition was observed in the control heart except for occasional positivity in the basement membrane of blood vessels. Then, the myocardial cell damage resembling the MAC positivity was evaluated by transmission electron microscopy, highlighting numerous and regular shaped lesions with diameters of 9–12 nm in the sarcolemma fragments of infarcted tissue. The authors confirmed these results in a later study, investigating the co-deposition in myocardial infarction of MAC with clusterin, a plasma glycoprotein involved in the regulation of MAC activation, reporting also the activity of this protein, as well as other regulators, in the clearance of the damaged tissue rather than in the protection of the myocardial cells from full assembly of the MAC [37]. 

Furthermore, Väkevä et al. [38] investigated the time course of the expression of factors (C1, C3, C8, and C9) involved in MAC activation in infarcted injuries analyzing a rat sample divided into eight groups on the basis of the time interval between coronary ligation and death. The immunofluorescence analysis revealed the early deposition of complement components foci in the areas supplied by the ligated artery in the 3-h infarction group. 

Another experimental study aiming to evaluate the time course of complement activation was performed by Sumitra et al. [39] using the immunohistochemistry for C5, C7, C8, and (s)C5b-9 and the ELISA analysis for (s)C5b-9 in serum and heart tissue. These investigations were performed on specimens belonging from rats with coronary artery ligation sacrificed at 1, 2, 4, 8, 16, and 32 h revealing the tissue immunopositivity of all markers and the significant increase of serum and myocardial (s)C5b-9 in the 8 h group. The authors also analyzed the ultrastructural change of tissue through a transmission electron microscope, highlighting the distortion of myofibrils, disruption of the cristae, and double membrane associated with deposition of amorphous dense particle and vacuole formation in mitochondria in the same group.

The time course of C5b-9 accumulation in myocardial infarction was experimentally analyzed by Mathey et al. [40] in rabbits in which the coronary was occluded for different periods between 30 minutes (min) and 29 h. The immunohistochemical staining for C5b-9 became increasingly positive after 5 h of occlusion and more intense at 12 h; the positivity was observed in the sarcolemma and intracellular space as in the arterial wall of infarcted and non-infarcted areas. The increase of C5b-9 was also studied by ELISA (normal concentration found at 102 ± 30 ng), highlighting differences in the concentration levels between ischemic and non-ischemic areas after 6 h and more. Furthermore, the authors investigated the effects of reperfusion in the second group of rabbits, revealing MAC immunopositivity after 15–30 min that become more intense at 3–4 h. 

In order to evaluate the mechanism of MAC deposition, Tada et al. [41] immunohistochemically studied the expression of C1q, C3q, MAC, and IgM, comparing with CD59 (MAC inhibitor) in autopsy heart sample with infarct age ranged between 3.5 h and 12 days. In myocardial infarction with a duration of 4 h or less, a focal distribution of complement components was observed in the cytoplasm of the left ventricular wall and interventricular septum cells, but the immunopositivity resulted in being more for C1q and C3q; MAC showed strong positivity in blood vessels. Then, a marked tissue deposition of MAC associated with the progressive CD59 depletion was highlighted in infarction aged 20 h and more. 

Ilczuk et al. [42] analyzed the complement components (C4d, C9, C1q, and C5b-9) and inhibitors (CD46, CD55, CD59, and factor H) to evaluate the contribution of the innate humoral activity of the nonspecific immune response. The study was performed on autopsy heart specimens with clear signs of acute myocardial infarction demonstrating the markers immunopositivity in all cases, as multiple myocardial cells staining in a necrotic zone with intensity from weak to marked. 

Complement activation after myocardial infarction was also studied by Yasojima et al. [43], detecting mRNAs and proteins for several complement components using PCR, Western blotting, and immunohistochemistry on autopsy myocardial tissue with recent and old infarction. All the investigations revealed a significant increase of complement products in the infarcted tissue and the activation of the classical pathway. Moreover, the authors suggested the complement components endogenous production by the human heart on the basis of the increased expression of complement mRNAs.

Ito et al. [44] investigated the influence of MAC on infarct size (by immunohistology staining with triphenyl tetrazolium chloride), reflow (by fluorescence staining with propidium iodide), and arrhythmogenesis on a rabbit sample composed by C6-competent and C6-deficient animals divided into two groups, respectively, with 30 min or 2 h of coronary artery occlusion followed by reperfusion. The study showed a smaller infarct size in C6-deficient rabbits of 30 min group compared to those C6-competent, while the extent of reflow was similar in both animal groups. In the group 2 h no differences, between C6-deficient and C6-competent were observed in infarct size, while the extent of reflow appeared to be markedly smaller in C6-competent rabbits. Another relevant consideration regarded the influence of C5b-9 in arrhythmogenesis, being observed severe arrhythmias in C6-competent rabbits of 30 min group.

The expression of C5b-9 in acute myocardial infarction was then studied by Nijmeijer et al. [45] in autopsy sample of myocardial tissue aiming to evaluate the relation between MAC and C reactive protein (CRP) in infarction (histologically staged as early phase, i.e., 0–12 h, PMN phase, i.e., 12 h–5 days, and chronic phase, i.e., 5–14 days), reinfarction (histologically staged as early phase and PMN phase) and reperfusion. The immunohistochemical analysis showed the deposition of MAC and CRP in sarcolemma, cross-striation, and cytosol of infarcted, re-infarcted, and reperfused tissue. The markers deposition resulted in being wider in re-infarcted tissue than in a single infarct in which the most extensive positivity was observed in the PMN phase. A larger immunopositivity was also highlighted in tissue reperfused by coronary artery bypass graft. 

Similar findings concerning the effects of reperfusion were reported by Böttiger et al. [46] investigating the activation of complement and PMN during cardio-pulmonary resuscitation (CPR) and after the restoration of spontaneous circulation (ROSC). C3a, (s)C5b-9, ELISA-analyzed PMN elastase, (s)P-selectin, and (s)ICAM-1 in the serum of patients who underwent CPR and in those with ROSC showed increasing levels both during CPR and for ≤48 h after ROSC. 

Bavia et al. [47] studied the complement system as an early marker for myocardial infarction clinical assessment. The analysis of the plasma levels of C3d and (s)C5b-9 showed higher concentration already at hospital admission of patients resulting in earlier respect to the classical myocardial necrosis markers (creatine kinase, creatine kinase-MB, myoglobin, and Troponin-I) in which higher concentrations were observed after 6 h. The main data of each article are summarized in Table 1.

### 3.2. Summary of Forensic Studies

Brinkmann et al. [48] analyzed the C5b-9 complex immunohistochemically, together with other markers (cellular and plasma proteins), in autopsy heart samples of cardiac deaths with or without coronary thrombosis, which were divided into two groups on the basis of the presence of histological evidence finding of infarction. The infarction group (presenting coagulative necrosis and contraction band necrosis) showing a marked loss of desmin and myoglobin, as well as a highly increasing of fibrinogen and C5b-9 in necrotic fibers, similar findings were observed in subjects with coronary thrombosis and without infarction even if the extension and the intensity of the immunoreactions were weaker. Even weaker reactions were observed in samples of subjects affected by coronary atherosclerosis without thrombosis and infarction. The investigations were also carried out in a control group composed of subjects who died for other causes and with known agony reporting the C5b-9 negativity.

Edston and Kawa [49] investigated the C5b-9 in sudden death with and without coronary artery disease and in a control group (hanging death). They reported the positivity in the arterial wall in 97% of coagulative necrosis areas and 65% of contraction band necrosis, highlighting that MAC positivity was found before the visible hematoxylin-eosin staining signs. Moreover, the authors showed cases of contraction bands without immunoreactions suggesting their agonal/artefactual origin. 

Thomsen et al. [50] tested the immunohistochemical expression of C5b-9 complex in cardiac autopsy material of subjects with myocardial infarction with an average survival time of 7–10 h postinfarction (showing limited histological evidence) compared to those samples of subjects with direct myocardial injuries (i.e., knife/gunshot wounds, cardiomyopathy, pericardial tamponade, etc.) and indirect myocardial lesions (i.e., mechanical asphyxia, exsanguination, anaphylactic shock, etc.). The analysis revealed the marker expression in the deep layer of arterial walls (internal positive control) and a specific and sensitive positivity only in myocardial infarction specimens.

Ortmann et al. [51] conducted another comparative study on the expression of cardiac antigens, plasma proteins and C5b-9 in cardiac sample belonging from subjects died for myocardial infarction, acute cardiac death and acute hypoxia (control group). They observed the depletion of the FABP (fatty acid-binding protein), troponin C and T, desmin and myoglobin, the deposition of the fibrinogen, fibronectin, and the complement complex C5b-9. The immunopositivity of MAC was found 30 min after the onset of symptoms. However, it highlighted an earlier depletion of cardiac antigens. Moreover, the C5b-9 expression seemed to be less affected by cardio-pulmonary resuscitation.

Similar findings were then described by Campobasso et al. [52] in a study carried out by immunohistochemistry on autopsy samples of acute myocardial infarction, coronary death, acute cardiac death, and acute traumatic death. Changes in expression of C5b-9, myoglobin, troponin-I, and fibronectin were observed in different grades in all groups, except for the control group in which there was the normal distribution of cardiac proteins associated with negative tissue staining for C5b-9 and fibronectin. The authors highlighted that C5b-9 is an earlier, sensitive, and specific marker of necrosis capable of demonstrating the damage in a small group of cells and/or single fibers in both coronary and acute cardiac death.

Kawamoto et al. [53] performed another comparative study of the expression of C5b-9, connexin 43 (Cx43), non-phosphorylated connexin 43 (npCx43), and zonula occludens-1 (ZO1) on autopsy sample revealing the immunopositivity for MAC in the 66.7% (n: 10/15) of the case with myocardial infarction and the 75% (n: 6/8) of the acute ischemic heart disease cases (without apparent myocardial necrosis). The immunostaining was observed in the myocyte cytoplasm of the left ventricle and interventricular septum, while negative resulted in the right ventricle. The control groups composed of mechanical asphyxia (n: 24) and drowning (n: 10) deaths were also negative at MAC staining.

The C5b-9 complex was also comparatively tested by Sabatasso et al. [54] in an experimental study performed on rats sacrificed at different time intervals after the artery coronary ligation. The immunohistochemical staining showed a scattered accumulation of MAC in single myocardial cells at 2 h point time group compared to the earliest detection of other molecules as Cx43 and JunB (15–30 min), cardiac troponins, myoglobin and fibronectin (1–2 h). 

Mondello et al. [55] compared C5b-9 to fibronectin and dystrophin in samples of hearts belonging from sudden cardiac death cases related to coronary atherosclerotic disease showing respectively clear signs of myocardial necrosis and no specific histological evidence of myocardial ischemia. Immunostaining for C5b-9 was observed in both groups, showing different intensity and spread, ranging from intense and diffuse to slight and/or patchy. Moreover, on the basis of the comparison of the marker features, the authors reported similar results about C5b-9 and dystrophin, whilst the overexpression of fibronectin was described as later. 

The specificity of C5b-9, together with fibronectin, was also tested by Fracasso et al. in two studies investigating the expression of the markers respectively in cases of fatal carbon monoxide intoxication and fatal ethanol intoxication. In the first study, C5b-9 resulted positive in all cases of myocardial infarction and negative in left ventricle taken from cases of CO intoxication and hanging in which, instead, fibronectin overexpression was observed [56]. The MAC negativity was also observed in the left ventricle of cases of ethanol intoxication [57].

Then, two studies investigating the susceptibility of C5b-9 to post mortem changes were selected by this literature review. Thomsen et al. [58] carried out a study to test the immunohistochemical expression of MAC in hearts belonging from four subjects with myocardial infarction clinically diagnosed and no specific histological signs, that were stored in a chamber at a temperature of 18–20° to proceed at specimen collections at different time intervals. The authors demonstrated intense and specific staining of infarcted tissue at a 0–170 h interval and less but specific staining of necrotic myocardium at 170–340 h samples; on the other hand, no specific staining was reported up to 390 h. These results were compared to histological findings observed in each interval groups supporting the marker usefulness in cases with a long post mortem interval in which the routine hematoxylin-eosin staining resulted to be inconclusive for the putrefactive phenomena. The same evidence was reported by Ortmann et al. [59] on specimens taken from hearts with extended acute myocardial infarction, incubated at room temperature of 16–20° for up to 8 weeks.

Moreover, a second part of the study was performed on samples of hearts taken from exhumations of cases with several findings indicating an acute myocardial infarction and cases with acute death and low grade of coronary atherosclerosis as a control group. In about half of the cases with presumed acute cardiac injuries, the C5b-9 positivity was observed, and the longest burial time was of 128 days. On the contrary, all the cases of the control group resulted in negative.

The main evidence described by each research group is reported in Table 2.

## 4. Discussion

The activation of the complement system is significantly related to myocardial damage due to ischemia and reperfusion, as demonstrated by evidence highlighting the reduction of ischemic myocardial tissue injury when depletion of complement components occurs, resulting in lowering neutrophils tissue infiltration [60,61]. The cause of the complement activation on cardiomyocytes during ischemia has not been clarified, but it was suggested that ischemia determines the plasma membrane damage resulting in the cellular loss of the capacity to inactivate the C3b components responsible for C5 activation and C5b-9 self-attach of the cell [62].

The damage mediated by complement can be caused directly or indirectly. The direct damage depends on the activation of complement factors themselves and MAC; the indirect damage is triggered by the activation of complement components that by acting as anaphylatoxins can mediate neutrophils activation and tissue infiltration and, thus, the production of toxic oxygen products and release of proteases [63,64]. Accordingly, MAC and neutrophils act in a coordinated manner to promote the degree of the irreversible ischemia/reperfusion injury [62]. 

In this context, MAC has an important role in mediating the direct myocardial tissue damage by the cytolytic form inducing the alteration of plasma membrane integrity/function and the transmembrane movement of electrolytes (accumulation of calcium and sodium, loss of potassium) and some proteins (i.e., creatine kinase) [63]. 

The deepening of the knowledge on the association between complement system activation and amplification of myocardial tissue damage induced several researchers to conduct studies to define better how and when the complement components act in this type of injuries, suggesting the possible contribution of specific inhibitory therapy to promote effective treatment of ischemic/reperfusion injury [24,65]. 

However, the pathophysiological/clinical research progress was accompanied and integrated by the development of prosperous post mortem/forensic works together, contributing to making out the complex mechanism of the complement system in myocardial ischemia/reperfusion injury. All the data reported in these studies support the usefulness of the analysis of complement components and, particularly, MAC in post mortem analysis of myocardial ischemia cases with a short time interval between the onset of the ischemic insult and death, in which the routine investigations result to be inconclusive. 

The analyzed studies contribute to evaluate the main features that a post mortem marker should have to consider its application useful and, thus, demonstrating sensibility, specificity, and stability. Obviously, in this context, also the method used for the marker analysis is important, having to be simple, reliable, and replicable. Immunohistochemistry seems to prove these features, resulting one of the most common investigation used in several forensic setting to assess the causes of death (i.e., sepsis and asphyxia) [66,67,68,69]. 

### 4.1. Complement Components for MAC Activation

Many studies included in the revision investigated several components factors belonging to the classical and alternative pathways in human or experimental samples of myocardial infarction, demonstrating that the MAC activation depends on both ways, even if some evidence suggested a prevalence of the classical pathway [34,36,43]. The components factors were observed only in ischemic or infarcted myocardial tissue, while, in control hearts, were negative, resulting exclusively in the artery walls positivity [36,37,42]. Moreover, these factors were analyzed in relation to the time interval of expression, revealing different findings: in human samples, the observed shortest time was about 4 h after the ischemic insult, while the longer time was after about 8 h [36,41]; in experimental samples, some researchers reported the expression of the components early, at 3 h, others confirmed the 8 h described in human hearts [38,39].

### 4.2. MAC Tissue Localization and Time of Expression

Numerous studies were carried out to analyze the specific involvement of MAC in myocardial ischemic injuries. The C5b-9 was analyzed by several methods aiming to evaluate localization and time of expression as well as the complement factors. 

First, both (m)C5b-9 and (s)C5b-9 forms were investigated by ELISA assay reporting the increase in serum of AMI patients and in autopsy heart specimens of subjects died for acute myocardial infarction, even if a more significant increase of the lytic form amount was described when the infarcted tissue was compared to non-pathologic tissue [35,47]. The same evidence was observed in experimental studies using serum and heart tissue taken from sacrificed animals with coronary artery ligation [39]; mainly, it was observed that differences in concentration between ischemic and non-ischemic areas were detectable after 6 h and more [40]. This increased expression led some authors to suggest that the degree of the activation of the complex could be related to the size of the myocardial necrotic areas [34].

The most relevant findings concerning both tissue localization and time interval of C5b-9 expression are derived from immunohistochemical studies carried out with forensic purposes.

The positivity of C5b-9 was observed in sarcolemma, cytosol, and interstitial space of ischemic/infarcted tissue and in artery walls of damaged and non-damaged tissue (positive internal control) [40,50,53,55]. Moreover, the marker was specifically investigated in contraction band necrosis (CBN), a type of histological damage that, even if associated with myocardial ischemia, cannot be considered as pathognomonic sign observed in other conditions as drowning, electrocution, cardiopulmonary resuscitation [70]. The MAC positivity was observed in CBN related to coronary artery disease, while it negatively resulted in cases in which the lesions seemed agonal or artefactual [49]. 

Studies investigating the temporal interval of expression were performed based on both routine histology evidence in cases with atherosclerotic coronary artery disease (from definite coagulative necrosis to unspecific signs as mild myofiber eosinophilia, elongation of sarcomeres and nuclei, wavy fibers and interstitial edema) and experimental studies in which the animals were killed after different time point from the artery ligation. Considering the “histological time”, the C5b-9 positivity was observed intense and diffuse in coagulative necrosis and, generally, slight and patchy in unspecific damage, this evidence supporting the analysis of MAC allows to identify the ischemic damage before it became histologically detectable [48,52,55]. A research group reported the C5b-9 deposition in cases with a circumstantial survival time of 30 min from the onset of the symptoms [51]. More specific data about the early detection of the marker belonged from experimental studies showing the antibody deposition after 2 h [54] or 5 h [40]. Another related aspect was represented by reperfusion affecting the MAC deposition on both times of detection and degree of expression: when reperfusion occurred, the immunopositivity was earlier (15–30 min) [40] and more intense and extensive [45]. Thus, it supports the role of C5b-9 in reperfusion injury occurring in the early phase of ischemia, determining the increasing of infarct size [44].

### 4.3. MAC Specificity

The MAC specificity was investigated comparing heart samples of subjects who died for myocardial ischemia/infarction to subjects with different causes of death. A study was carried out for this purpose differentiating the death due to myocardial infarction and the death in which the myocardial damage occurred directly (i.e., knife or gunshot injuries, pericardial tamponade) or indirectly (i.e., exsanguination, anaphylactic shock); the immunopositivity was specific and sensitive only in cases of myocardial infarction [50]. The marker was also negatively described in left ventricle specimens belonging from hanging, drowning, acute traumatic death, carbon monoxide intoxication, and ethanol intoxication [51,52,53,55,56,57].

### 4.4. MAC Stability

The terminal complement complex was studied concerning the post mortem stability because it is well known that autolysis and putrefaction determine the cellular and tissue degradation affecting the reliability of the histological findings and, possibly, the expression of molecules immunohistochemically (or by other techniques as ELISA, forensic biochemistry) analyzed [23]. The most important data were provided by the immunohistochemical analysis of C5b-9 in exhumated bodies in which the immunopositivity was observed at a burial time of 128 days [59]. On the other hand, two experimental studies provided discordant evidence reporting an immunopositivity until 390 h and 8 weeks, respectively; the cause of this cannot define even if the difference in the method sampling might have a role because the first one was performed storing the whole heart at 18–20 °C, the second one incubating heart specimens at 16–20 °C [58,59]. Moreover, useful evidence emerged from the analysis of complement components mRNAs reporting little differences in the quality and the levels detected by RT-PCR in post mortem intervals varying from 17 to 144 h [43]. 

Another factor regarding the marker stability is the performed cardiopulmonary resuscitation (CPR), whose effects are strictly related to myocardial reperfusion damage determining complement activation and C5b-9 increasing early, as above described [40,44,45]. A study carried out on serum of patients who underwent to resuscitation after cardiac arrest for non-traumatic causes showed the increasing of (s)C5b-9 levels during CPR and also for ≤48 h after the restoration of spontaneous circulation [46], suggesting that CPR might affect the reliability of the MAC when used for the post mortem diagnosis of myocardial ischemia/infarction. On the contrary, a post mortem study by immunohistochemistry showed C5b-9 negativity in 4/5 cases of hanging with CPR [51].

### 4.5. Comparison with Other Markers

Finally, the revision highlighted the analysis of C5b-9 expression compared to other molecules as cellular proteins and plasma proteins [48,51,52,53,54,55]. These comparative analyses revealed that cellular proteins such as troponins, myoglobin, and connexin 43 showed an earlier alteration of the regular expression than C5b-9 deposition, suggesting remarkable usefulness for the diagnosis of early myocardial ischemia [51,54]. On the other hand, these cellular markers were described with limits related to the influence of CPR and post mortem phenomena on their expression [23,59].

## 5. Conclusions

The data emerging from this literature review describe the main features of the complement system and MAC in myocardial ischemic/reperfusion injury by the evidence reported in several studies aiming to explain the functions of the MAC and clarify how and when the complement complex contribute to this type of damage. These findings were analyzed with a forensic perspective to define the characteristics of MAC use in post mortem diagnosis of myocardial ischemia/infarction. Several studies carried out on this issue support the MAC usefulness as sensible and specific post mortem marker of myocardial infarction also representing an essential tool for cases with very short survival time after the ischemic insult, as a marker of early myocardial ischemia. Most of the studies carried out by immunohistochemical analysis of the C5b-9 marker have demonstrated the simplicity and reproducibility of the method itself. Moreover, C5b-9 represents the only marker tested for post mortem stability showing positive results that support the value in advanced autolysis or putrefaction [22,23,71]. Thus, the analysis of C5b-9 in forensic cases of suspected myocardial ischemia can be recommended. However, further studies on other selected (inflammatory) molecules assessing their specificity and stability to incorporate them, together with C5b-9, into routine diagnostics, and creating an effective and reliable panel of markers to confirm the accuracy of the diagnostic results.

## Figures and Tables

**Table 1:** Summary of the main data resulting from analyzed pathophysiological/clinical studies.

Authors	Sample	Time Interval	Type of Damage	Marker	Method	Results
Yasuda et al. [34]	Patients (31 AMI, 17 UAP, 19 SAP)	≤ 24 h	ischemia	iC3b, C3d, C4d, Ba, Bb, (s)C5b-9	ELISA	↑ (s)C5b-9 in AMI ↑ others in AMI and UAP
Hugo et al. [35]	36 autopsies	1–8 d	ischemia	(m) and (s) C5b-9	ELISA	↑↑ of (m)C5b-9 ↑ of (s)C5b-9
Väkevä et al. [36]	13 autopsies	2.5 h–14 d	ischemia	C1q, C3c, C3d, C4, C5, C6, C8, C9, C5b-9	IFL	↑ at 8 h and more
Väkevä et al. [37]	10 autopsies	8 h–14 d	ischemia	clusterin, C5b-9, C1q, C3d, C4, C9	IFL	↑ at 8 h and more
Väkevä et al. [38]	36 rats	1, 2, 3, 6, 24, 72 h	ischemia	CD59, C1, C3, C8, C9	IFL	↑ C3 at 2 h ↑ C1, C8, C9 at 3 h ↓ CD59 from 6 to 72 h
Sumitra et al. [39]	36 rats	1, 2, 4, 8, 16, 32 h	ischemia	(s) C5b-9	ELISA	↑ at 8 h and more
				(s) C5b-9, C5, C6, C7, C8, C9	IHC	
Mathey et al. [40]	17 rabbits	30 min, 1.5–3, 5–6, 12–17, 22–29 h	ischemia	(m) C5b-9	ELISA	↑ at 6 h
					IHC	↑ at 5 h
	23 rabbits	30 min, 1, 1.5, 2, 3, 5 h	reperfusion		ELISA	↑ within 1 h
					IHC	↑ at 15–30 min
Tada et al. [41]	9 autopsies	3.5 h–12 d	ischemia	C1q, C3d, MAC, IgM, CD59	IHC	↑ C factors at 4 h ↓ CD59 at 20 h
Ilczuck et al. [42]	50 autopsies	n.d. (clinical and histological diagnosis)	ischemia	C1q, C4d, C9, CD45, CD55, CD59, fH	IHC	↑ (all markers)
Yasojima et al. [43]	AMI (recent and old) from 5 autopsies	n.d. (clinical and/or histological diagnosis)	ischemia	C1q, C1r, C1s, C2, C3, C4, C5, C6, C7, C8, C9, C5b-9	RT-PCR (mRNA)	↑ (all markers) mRNA stability until 132 h post mortem
					WB	
					IHC	
Ito et al. [44]	28 rabbits (C6 competent), 18 rabbits (C6 deficient)	30 min, 2 h	ischemia	C5b-9	IHC	↑ at 30 min C6 competent
			reperfusion			↑ at 2 h in both
Nijneijer et al. [45]	76 autopsies	0–12 h, 12 h–5 d, 5–14 d	ischemia	PCR, C3d, C5b-9	IHC	↑ (all markers)
			reinfarction			↑↑ (all markers)
			reperfusion			↑↑ (all markers)
Böttiger et al. [46]	21 patients with CPR and ROSC (n:7)	15, 30 min (CPR) 30 min, 2, 8, 24, 48, 72 h, 7 d (ROSC)	reperfusion	C3a, (s)C5b-9	ELISA	↑ during CPR ↑ after ROSC (≤48 h)
Bavia et al. [47]	17 AMI patients	> 30 min	ischemia	CK, CK.MB, My, Tr-I, C3d, (s)C5b-9	ELISA	↑ C3d and (s)C5b-9 at hospital admission

AMI: acute myocardial infarction; UAP: unstable angina pectoris; SAP: stable angina pectoris; CPR: cardiopulmonary resuscitation; ROSC: restoration of spontaneous circulation; fH: factor H; CRP: C reactive protein; CK: creatin kinase; CK-MB: creatin kinase MB; My: myoglobin; Tr-I: troponin I; IHC: immunohistochemistry; IFL: immunofluorescence; min: minutes; h: hours; ↑: increased expression; ↑↑: very increased expression; ↓: reduced expression; n.d.: not defined.

**Table 2:** Summary of the main data resulting from the analyzed forensic studies.

Authors	Sample	Time Interval	Marker	Method	Results	Other Findings
Brinkmann et al. [48]	autopsies: AMI (I group: 15), coronary thrombosis without infarction (II group: 11), only coronary atherosclerosis (III group: 9)	histologically: from detectable coagulative necrosis to weak or no signs	Desmin, myoglobin, fibrinogen, C5b-9	IHC	↑ fibrinogen, C5b-9 in I-II groups ↓ desmin, myoglobin in I-II groups	Negativity of C5b-9 in control group (gunshot, drowning hanging, SIDS, etc)
Edston and Kawa [49]	autopsies: SD with CAD (I group: 44), SUD without CAD (II group: 25)	histologically: from detectable coagulative necrosis to weak or no signs	C5b-9	IHC	↑ in coagulative necrosis and in some areas of CBN	Negativity of C5b-9 in agonal/artefactual CBN and in control group (hangings)
Thomsen et al. [50]	autopsies: AMI (n:n.d.), direct myocardial injury (n:28), indirect myocardial lesions (n:38)	7–10 h (AMI)	C5b-9	IHC	↑ in AMI group, negative in direct and indirect injuries	
Ortmann et al. [51]	autopsies: AMI (group I: 8), SCD without histological necrosis (group II: 8)	histologically: from detectable coagulative necrosis to weak or no signs	Troponins, FABP, CD59, myoglobin, desmin, C5b-9, fibrinogen, fibronectin	IHC	↑ C5b-9 in both groups ↓↓ cellular proteins	Negativity of C5b-9 in control group (hanging), also in 4/5 cases with CPR
Campobasso et al. [52]	autopsies: AMI (group I: 6), coronary death (group II: 4), acute cardiac death (group III: 8)	group II < 1 h, group III < 8–10 h	Troponin I, myoglobin, fibronectin, C5b-9	IHC	↑ C5b-9 in group I ↑↑ C5b-9 in group III ↓↓ My in group II ↓↓↓ Tr and M in group III	Negativity of C5b-9 in control group (acute traumatic death)
Kawamoto et al. [53]	autopsies: MI (group I: 15), acute ischemic heart disease (group 2: 8)	histologically: from detectable coagulative necrosis to weak or no signs	Cx43, npCx43, ZO-1, C5b-9	IHC	↑ C5b-9 in group I and II ↑ npCx43 in group I and II	Negativity of C5b-9 in control group (hanging, drowning)
Sabatasso et al. [54]	44 rats	5, 15, 30 min, 1, 2, 4, 7, 12, 24 h	Cx43, fibronectin, troponins, myoglobin, C5b-9	IHC	↑ Cx43 at 15–30 min ↑ Tr, My, at 1 h ↑ C5b-9 at 2 h	
Mondello et al. [55]	autopsies: MI (n:12), EMI (12)	histologically: from detectable coagulative necrosis to weak or no signs	Dystrophin, fibronectin, C5b-9	IHC	↑ C5b-9 in EMI ↑ FN in EMI ↓ Dys in EMI	Negativity of C5b-9 in control group (acute mechanical asphyxiation); C5b-9 and Dys earlier than FN
Fracasso el al. [56]	autopsies: AMI (n: 25), CO intoxication (n: 26), hanging (n: 23)	AMI histologically detectable	C5b-9, fibronectin	IHC	↑ C5b-9 in AMI C5b-9 negative in CO intoxication and hanging ↑ FN in all groups	//
Fracasso et al. [57]	autopsies: AMI (n: 18), alcohol intoxication (n: 19), hanging (n: 15), pulmonary thromboembolism (n:26)	AMI histologically detectable	C5b-9, fibronectin	IHC	↑ C5b-9 in LV of AMI C5b-9 negative in LV of ET, PT and hanging ↑ FN in LV of all groups	//
Thomsen et al. [58]	autopsies: AMI (n:4) PMI from 0 to 480 h	AMI macroscopically and histologically detectable	(m)C5b-9	IHC	C5b-9 detectable until 340 h post mortem	//
Ortmann et al. [59]	autopsies: AMI (n:3), hanging (n:3) PMI from 0 to 8 w	AMI histologically detectable	C5b-9, fibronectin, fibrinogen, desmin, troponin C, FABP, myoglobin	IHC	C5b-9 detectable until 8 w post mortem Other proteins artefacs after 1 w	//
	exumation: AMI (n:20), controls (n:6) PMI from 10 to 929 d	supposed AMI by clinical records, CAD			C5b-9 detectable at 128 d post mortem	

AMI: acute myocardial infarction; SD: sudden death; CAD: coronary artery disease; SUD: sudden unexpected death; SCD: sudden cardiac death; EMI: early myocardial ischemia; CBN: contraction band necrosis; PMI: post mortem interval; FABP: fatty acid-binding protein; Cx43: connexin 43; np Cx43: non-phosphorylated connexin 43; ZO-1: zonula occludens-1; min: minutes; h: hours; ↑: increased expression; ↑↑: very increased expression; ↓: reduced expression; ↓↓: very reduced expression; ↓↓↓: loss of expression.
